# Intra- and inter-observer reliability of girth measurements of the neck, chest, and abdomen in dogs

**DOI:** 10.3389/fvets.2025.1546951

**Published:** 2025-03-13

**Authors:** Josefin Söder, Ludvig Ehnberg, Erica Löfberg, Katja Höglund, Anna Bergh

**Affiliations:** ^1^Department of Clinical Sciences, Faculty of Veterinary Medicine and Animal Science, Swedish University of Agricultural Sciences, Uppsala, Sweden; ^2^Department of Animal Biosciences, Faculty of Veterinary Medicine and Animal Science, Swedish University of Agricultural Sciences, Uppsala, Sweden

**Keywords:** body composition, body condition assessment, BCS, canine, dynamometer, tape measure, overweight

## Abstract

**Objective:**

This study aimed to assess intra- and inter-observer reliability of neck, chest, and abdominal girth measurements in dogs and to compare these measurements made with a measuring tape, equipped with or without a dynamometer.

**Methods:**

The locations of the middle neck, cranial and widest chest, and cranial and caudal abdomen were measured individually by two observers in 16 dogs standing squarely at an examination table. Girth measurements were performed in triplicate with the other observer recording the data. All dogs underwent evaluation using a measuring tape equipped with a spring dynamometer, while a subgroup (*n* = 8) was also evaluated with a measuring tape without the dynamometer. Intraclass correlation coefficients (ICCs), with a 95% confidence interval (CI), were computed to assess the intra- and inter-observer reliability for the measurements made with the measuring tape equipped with a spring dynamometer. Pearson’s correlations (*r*) were used to compare the two methods: girth measurements performed with and without the dynamometer.

**Results:**

Girth measurements at all locations demonstrated high intra-observer (0.967–0.999) and inter-observer (0.985–0.995) reliability. The correlations between measurements made with and without the dynamometer were high (*r* ≥ 0.996, *p* < 0.0001). Numerically higher girth values with numerically lower precision were recorded using the tape measure without the dynamometer, but only the girth of the cranial abdomen differed significantly between methods (*p* = 0.04).

**Conclusion and clinical importance:**

Girth measurements were reliable across all locations, particularly in the cranial chest and caudal abdomen, which exhibited high precision both within and between the two observers. A tape measure loaded with a dynamometer is recommended, as measurements recorded with a tape measure only showed a tendency of higher girth values with lower precision. Future research should evaluate neck, chest, and abdominal girth measurements in overweight canine patients, as well as the usefulness of the method as a complement to clinical body condition assessment for tracking changes in body composition.

## Introduction

Objective outcome measures are important in veterinary medicine for accurate diagnosis and for assessing treatment efficacy. Ideal measures demonstrate low variability and high reliability when compared within and between observers, showing high intra- and inter-observer reliability (1). One way of assessing different body measures is by using a measuring tape. Girth measurement of limbs has been suggested as an objective, quick, and reliable method to indirectly evaluate the muscle mass of dogs (2–5), but the reliability of assessing other body parts using a measuring tape is unknown. Overweight in dogs is a common issue, affecting approximately 40% of the Swedish dog population (6, 7) and 60% of dogs in the United States and the United Kingdom (8, 9). Assessment of canine overweight is commonly performed with a joint approach of using a body condition score (BCS) system (10) and registering of body weight (11). The BCS system has good intra- and inter-reliability (10, 12, 13) but is regarded as a semi-objective method that primarily correlates with total body fat rather than muscle mass (10). In addition, BCS assessment requires training (6, 14, 15), and dog owners often underestimate overweight status (6, 16–18). Therefore, veterinary staff and dog owners need objective and simple yet reliable methods for evaluating body composition, including both fat and muscle mass (13, 19), which can be a value addition to the clinical BCS assessment.

There is a general need to maintain the ideal body condition of all dogs and to diagnose and treat overweight for good canine health and welfare (8, 20), as overweight increases the risk for co-mortality and morbidity (21–25). In addition, underestimating overweight status may increase the risk of further weight gain. Fat and muscle mass can be evaluated by advanced, objective methods such as dual-energy X-ray absorptiometry (DEXA); however, this method is expensive and requires sedation or anesthesia of the dog (13). Despite receiving the same score on the BCS scale, different dog breeds may vary in their overall fat percentage evaluated by DEXA (19), highlighting the need for more refined clinically applicable methods. Weight loss interventions include caloric restriction and/or suggestions for increased physical activity (26–30). During these interventions, dogs are expected to alter their body fat and muscle mass proportion to reduce fat mass while maintaining muscle mass during weight loss. Chest and abdominal girth measurements have been shown to decrease significantly during weight loss interventions with or without the addition of increased physical activity (26–28). The subcutaneous fat layer over the ribs is particularly important, as this parameter is vital for canine BCS assessment (10, 31). The fat layer in this location is represented by chest girth measurements, which have previously been shown to be associated with BCS (28, 32). The abdominal girth is a measurement location that includes intraabdominal fat, subcutaneous fat, and the muscle mass of the lumbar region (28, 31, 33). These components comprise parameters evaluated in a BCS assessment (10). In addition, the neck girth would also be considered for measurements, as obese dogs accumulate fat deposits on the neck (10).

Several factors can influence girth measurements made with a measuring tape. The performance of the observer, the impact of the measurement device, and the conformation of the measurement site have been previously demonstrated to influence reliability (2, 3, 5, 34–36). Two previous canine studies have indicated high intra-observer reliability of girth measurements of the chest and abdomen (28, 37). However, to our knowledge, girth measurements made with a measuring tape for the neck, chest, and abdomen have not been previously evaluated for both intra- and inter-observer reliability in dogs. To reduce the impact of the obesity epidemic among pet dogs, objective, reliable, and simple evaluation methods are essential for clinical practice. Therefore, the aim of this study was to assess the intra- and inter-observer reliability of neck, chest, and abdominal girth measurements in dogs and to compare these measurements made with a measuring tape, equipped with or without a dynamometer.

## Materials and methods

### Ethics approval and owner consent

The study was approved by the Ethics Committee for Animal Experiments, Uppsala, Sweden (Dnr 5.8.18–12,184/2023). All dog owners provided written consent before their dogs participated in the study. All dogs participated in the study alongside their owners or handlers.

### Recruitment of study population

Dog owners in the Uppsala region were invited by personal contacts to participate with their dogs in this study. Dogs above 1 year of age were included as a convenience sample on a non-randomized basis and were selected to represent small, medium, and large breeds as well as a variety of diverse body types among breeds. Moreover, dogs were included to represent a broad spectrum of body conditions, ranging from underweight to obesity. The exclusion criteria included known aggression or shyness in dogs, as these factors could influence their ability to participate in the study.

### Clinical data collection

Each dog was allowed to become familiar with the research setting and environment, and was then placed on an examination table in a standing position. If needed, the position of the dog was adjusted to stand squarely on the table, with its paws positioned symmetrically. The evaluations of this study were performed on awake, standing dogs where the fur was left untrimmed to replicate conditions in a clinical situation. All girth measurements were performed by two veterinary nursing students in their final semester of their educational program. The two students had no prior experience of girth measurements using a measuring tape on dogs, but they received training from their supervisor on how to use the method before the start of the data collection. During the same training session, the supervisor and both students agreed on the exact anatomical locations to be used for the girth measurements in the study. Henceforth, students will be referred to as “observer 1” and “observer 2”.

In this study, the observers were blinded to the girth measurements made, as the observer making the measurement focused on the dynamometer and instructed the other observer to read the value on the measuring tape when the correct end force was applied according to the dynamometer. The observer read the value aloud and the supervisor recorded it in a protocol. Data were collected from a dog cohort using a non-stretchable measuring tape equipped with a custom-made mechanical spring dynamometer. Additionally, a subgroup of the cohort was evaluated using a non-stretchable measuring tape alone (without a dynamometer). The subgroup of dogs was evaluated at the end of the 4-month study. The sequence of measurements made with the measuring tape, both with and without the dynamometer, was randomized within the subgroup of dogs. The observers were unaware of their initial results when conducting the repeated evaluation in the subgroup.

#### Bodyweight and body condition score

Bodyweight was measured to the nearest 0.1 kg using a Kruuse Scale 250 digital veterinary scale (Jørgen Kruuse A/S Langeskov, Denmark). A veterinarian specialized in BCS assessment (JS) evaluated the BCS in all dogs. The body condition assessments were conducted through visual inspection and palpation of areas over the ribs, waist and abdominal line, neck, and base of the tail, following the guidelines from the World Small Animal Veterinary Association (WSAVA) based on the validated 9-point BCS scale established by Laflamme (7). According to this scale, a BCS of 1–3 indicates underweight, 4–5 is ideal weight, 6 indicates slight overweight, 7 indicates overweight, and 8–9 indicates obesity. The bodyweight and BCS assessments were collected as single measurements.

#### Girth measurements of the neck, chest, and abdomen

**Figure 1:**
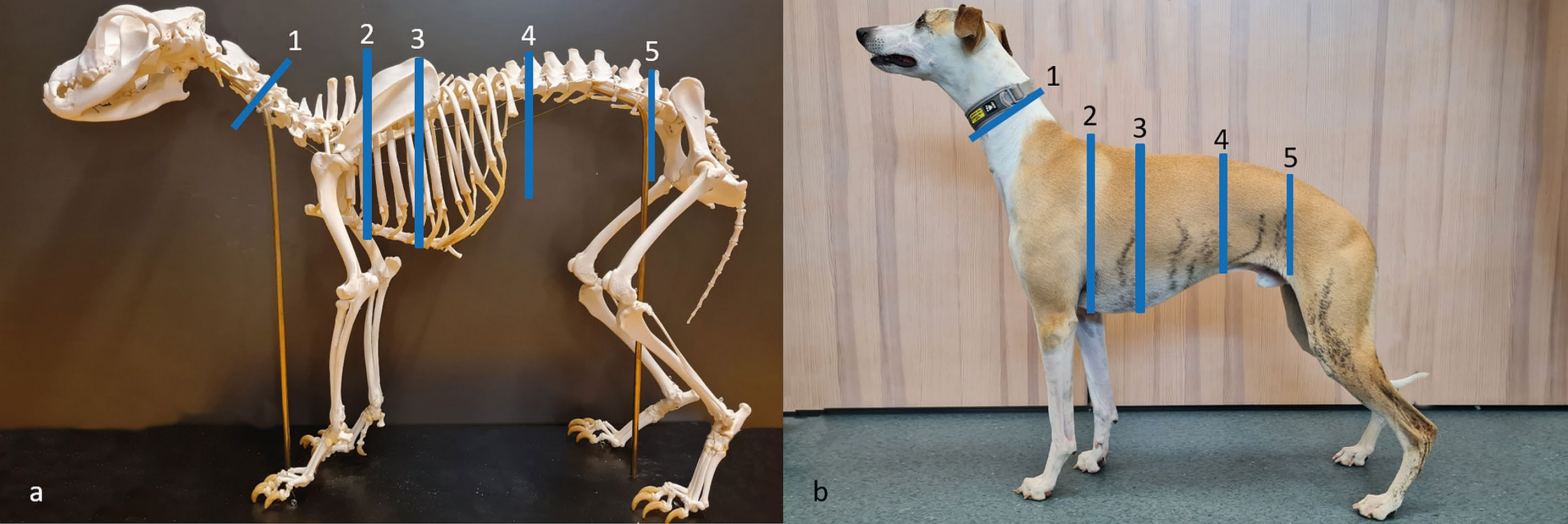
Anatomical locations for girth measurements on the neck, chest, and abdomen. All measurements were made in triplicate using a dynamometer attached to the measuring tape, as well as with a measuring tape alone without a dynamometer for a subgroup across five locations: 1: middle neck (half the distance from the crista nuchae to the cranioproximal crest of the scapula); 2: cranial chest (in the axilla); 3: widest chest (visually assessed from above); 4: cranial abdomen (caudal of the last rib); and 5: caudal abdomen (cranial of ileum). (a) Dog skeleton from the historic anatomical theater, Swedish University of Agricultural Sciences; (b) Whippet participating in the study. Photos and picture editing: Josefin Söder.

Figure 1 shows the markings of the anatomical locations on the neck, chest, and abdomen used for girth measurements in the study. The girth of the neck was measured at one location: halfway between the crista nuchae on the skull to the cranioproximal crest of the scapula, where the crista nuchae was palpated in the sagittal plane with the head in a neutral position. The halfway point was determined by palpation and visual estimation. The girth of the chest was measured at two locations: directly caudal to the elbow in the axilla and at the widest part of the chest evaluated visually from above. The girths of the abdomen were measured at two locations: directly caudal of the last rib and directly cranial of the cranial crest of the ileum at the pelvic bone. Each location was measured (in millimeters) in triplicate. The measuring tape was pulled vertically with a force of 0.3 kg (3 N) and was released from the measurement site between each individual measurement. The anatomical locations were measured in the order of 1–5 (Figure 1) in each dog.

### Data processing and statistical analyses

#### Data processing

Microsoft Excel, GraphPad Prism 5.0 (GraphPad Prism Software, Inc., San Diego, CA, United States) and Boston, United States: RStudio (2023.12.0 + 369 Posit Software, PBC) were used for data processing and statistical analyses. D’Agostino and Pearson’s omnibus normality tests were used to evaluate the normal distribution of the data. All data were normally distributed except for the girth measurements in the subgroup of dogs. The results are presented as mean value ± standard deviation (SD), except when expressing the precision of measurements, where standard error of mean (SEM) from the triplicates is used. The means of the triplicate methods were used to compare the girth measurements, looking at the differences between the observers and also between the measurements made with and without a dynamometer for each observer. The means of the calculated SEMs were used to compare the precision between observers and also between the measurements made with and without a dynamometer for each observer. The threshold for statistical significance was set at a *p-value of* < 0.05 for all analyses. See raw data in Supplementary File 1.

#### Paired analyses

Paired *t*-tests were used to compare the girth measurements between observers at each anatomical location, using the means of the triplicates of all dogs in the analyses. A Wilcoxon signed rank test was used to compare the girth measurements made with and without a dynamometer within the subgroup, at each anatomical location, according to each observer, using the means of the triplicates in the analyses.

#### Calculations of intra- and inter-observer reliability

The libraries “tidyverse” and “ICC” in RStudio were used to calculate the intraclass correlation coefficients (ICCs) with the 95% confidence interval (CI) for measurements performed with the dynamometer. The reliability within the observer (intra-observer reliability) was calculated from the individual triplicates (1, 2, and 3) for each observer at each anatomical location (middle neck, cranial chest, widest chest, cranial abdomen, and caudal abdomen). The reliability between observers (inter-observer reliability) was calculated based on the means of the triplicates from both observers 1 and 2 at each anatomical location. The correlation between measurements made with and without the dynamometer was analyzed with Pearson’s correlations (*r*), calculated from the means of the triplicates for each observer, with all anatomical locations combined. The values were plotted for the visualization of agreement. The interpretations of all ICCs were based on previously established levels of reliability: high reliability, 0.90–0.99; good reliability, 0.80–0.89; fair reliability, 0.70–0.79; moderate reliability, 0.69–0.59; and poor reliability, <0.59 (3).

## Results

### Description of dog population

In this study, a total of 16 dogs were included. For one of the dogs, the measurements of the middle neck were excluded due to the temperament of the dog, while all other dogs were evaluated with a measuring tape equipped with a dynamometer at all of the anatomical locations. Eight of the 16 dogs were additionally evaluated with a measuring tape alone, equipped without a dynamometer. A total of 714 recordings were made.

The 16 dogs included in this study represented a variety of small, medium, and large breeds, with a total of 13 different pure breeds as well as mixed breeds. The breeds included were as follows: mixed breed (*n* = 2), Borzoi (*n* = 1), Short haired Dachshund (*n* = 1), Eurasier (*n* = 1), Flat-coated Retriever (*n* = 1), Gordon Setter (*n* = 1), Jack Russell Terrier (*n* = 1), Labrador Retriever (*n* = 2), Lagotto Romagnolo (*n* = 1), Miniature Schnauzer (*n* = 1), Rottweiler (*n* = 1), Shetland Sheepdog (*n* = 1), Swedish Vallhund (*n* = 1), and Whippet (*n* = 1). The descriptive data of the dog population and its subgroups are presented in Table 1.

**Table 1:** Descriptive data of the dog population divided into the whole cohort of dogs and the subgroup of the dog cohort.

	Whole dog cohort (*n* = 16)	Subgroup of the dog cohort (*n* = 8)
Parameter	Mean ± SD	Mean ± SD
	Median (Range)	Median (Range)
Age (years)	5.7 *±* 2.3	6.6 *±* 2.5
	5 (3–10)	6 (4–10)
Bodyweight (kg)	21.2 *±* 13.3	18.2 *±* 14.2
	20 (5.5–50.0)	12.5 (5.5–50.0)
BCS (scale 1–9)	4.8 *±* 1.2	5.3 *±* 1.4
	4.5 (3–8)	5 (4–8)
BCS (scale 1–9)	Number	Number
3 (underweight)	1	0
4–5 (normal weight)	12	5
6 (slight overweight)	2	2
7 (overweight)	0	0
8 (obese)	1	1
Sex	Number	Number
Male (of which neutered)	9 (5)	5 (4)
Female (of which spayed)	7 (2)	3 (1)

BCS, Body condition score; SD, standard deviation. The whole cohort of dogs (*n* = 16) was evaluated using a measuring tape equipped with a dynamometer. The subgroup of the dog cohort (*n* = 8) was evaluated using a measuring tape equipped with a dynamometer as well as a measuring tape alone, in a randomized order.

### Girth measurements performed with a measuring tape equipped with a dynamometer

Observers 1 and 2 showed similar recorded mean girths for the different anatomical locations, except for the locations of the “cranial chest” and “caudal abdomen,” which showed significantly higher mean girths for observer 2 compared to observer 1 (Table 2). Observer 2 displayed higher means of SEM ± SD for all anatomical locations (Table 2), indicating a generally lower precision in measurements performed compared to observer 1. The anatomical location demonstrating the highest precision in measurements (the lowest means of SEM) was the “cranial chest” for both observers. Conversely, the anatomical location showing the lowest precision of measurements (the highest means of SEM) was the location of the “widest chest” for both observers (Table 2). The proportional size of the mean measurement error (the size of SEM in relation to the mean girth value at each location) was highest at the location of the “middle neck” (0.8–1%) and lowest at the location of the “cranial chest” (0.2–0.5%) for both observers.

**Table 2:** Comparisons among observers making girth measurements performed with a measuring tape equipped with a dynamometer.

	Observer 1		Observer 2		
Location	Girth (cm)	Precision of measurements (cm)	Girth (cm)	Precision of measurements (cm)	Difference in girth
	Mean ± SD	Mean SEM ± SD	Mean ± SD	Mean SEM ± SD	*p*-value
Middle neck	35.7 ± 9.6	0.30 ± 0.27	36.1 ± 9.1	0.54 ± 0.82	0.38
Cranial chest	63.0 ± 16.0	0.14 ± 0.09	64.2 ± 16.4	0.31 ± 0.22	0.0006
Widest chest	61.2 ± 15.5	0.35 ± 0.24	60.8 ± 15.2	0.60 ± 0.56	0.35
Cranial abdomen	50.8 ± 15.0	0.34 ± 0.23	50.9 ± 15.0	0.44 ± 0.36	0.87
Caudal abdomen	48.3 ± 14.2	0.23 ± 0.16	49.8 ± 15.0	0.45 ± 0.36	0.007

SD, standard deviation; SEM, Standard error of mean. The table presents data from all 16 dogs (15 dogs for the location of the “middle neck”). Paired *t*- tests were used to compare the girth measurements between observers at each location, where the means of the triplicates were used in the analyses. A *p*- value of < 0.05 was considered significant.

#### Intra- and inter-observer reliability

The girth measurements at all anatomical locations showed high intra-observer (0.967–0.999) and inter-observer (0.985–0.995) reliability according to the calculated ICCs, as summarized in Table 3. Observer 1 exhibited high intra-observer reliability across all anatomical locations, whereas observer 2 displayed slightly lower, yet still high, intra-observer reliability. Additionally, observer 2 exhibited wider CIs for all anatomical locations compared to observer 1, confirming the generally lower precision of measurements of observer 2. Observer 1 demonstrated the highest intra-observer reliability at the locations of the “cranial chest” and “caudal abdomen” (Table 3). Inter-observer reliability was high across all anatomical locations. The locations of the “cranial chest” and the “caudal abdomen” exhibited the highest inter-observer reliability, whereas the location of the “middle neck” displayed the lowest inter-observer reliability (Table 3).

**Table 3:** Intra- and inter-observer reliability of girth measurements performed with a measuring tape equipped with a dynamometer.

Location	Intra-observer ICC (95% CI)		Inter-observer ICC (95% CI)
	Observer 1	Observer 2	
Middle neck	0.995 (0.988–0.999)	0.967 (0.926–0.988)	0.985 (0.956–0.995)
Cranial chest	0.999 (0.999–1.000)	0.998 (0.996–0.999)	0.995 (0.986–0.998)
Widest chest	0.998 (0.995–0.999)	0.992 (0.981–0.997)	0.994 (0.983–0.998)
Cranial abdomen	0.998 (0.995–0.999)	0.996 (0.991–0.998)	0.988 (0.967–0.996)
Caudal abdomen	0.999 (0.998–1.000)	0.996 (0.990–0.998)	0.995 (0.986–0.998)

ICC, Intraclass correlation coefficient; CI, confidence interval. All individual observations (triplicates 1, 2, and 3) of girth measurements were included in the analyses of intra-observer reliability, while the mean of the observations (triplicates 1, 2, and 3) was used for analyses of inter-observer reliability. Analyses were conducted with a 95% confidence interval on data from 16 dogs (15 dogs for the location of the “middle neck”).

### Comparisons of girth measurements performed with and without a dynamometer attached to the measuring tape

Overall, a few significant differences were found between girth measurements performed with a measuring tape equipped with a dynamometer and a measuring tape alone (Table 4). However, both observers showed a tendency to higher girth values when measurements were performed alone with a measuring tape. However, only the location of the “cranial abdomen” differed significantly between methods for observer 2 (Table 4). Observer 1 demonstrated higher precision of measurements (lower means of SEM) than observer 2 at nearly all anatomical locations for measurements performed both with and without the dynamometer (Table 4). However, the differences were not significant (*p* ≥ 0.06), and the precision varied across different anatomical locations and among observers (Table 4). For all locations pooled, both observers showed numerically higher precision (lower means of SEM ± SD) for measurements performed with a measuring tape equipped with a dynamometer compared to measurements performed with a measuring tape alone (observer 1: 0.23 ± 0.17 versus 0.30 ± 0.20. Observer 2: 0.32 ± 0.19 versus 0.36 ± 0.26).

**Table 4:** Comparisons for each observer of girth measurements made with and without a dynamometer attached to the measuring tape.

Observer 1					
	With dynamometer		Measuring tape alone		
Location	Girth (cm)	Precision of measurements (cm)	Girth (cm)	Precision of measurements (cm)	Difference in girth
	Mean ± SD	Mean SEM ± SD	Mean ± SD	Mean SEM ± SD	*p*-value
Middle neck	34.4 ± 10.6	0.33 ± 0.30	34.2 ± 10.1	0.26 ± 0.16	0.74
Cranial chest	59.9 ± 16.8	0.19 ± 0.09	60.2 ± 16.9	0.27 ± 0.21	0.40
Widest chest	59.1 ± 16.5	0.19 ± 0.09	58.9 ± 15.9	0.41 ± 0.13	0.84
Cranial abdomen	49.7 ± 17.3	0.28 ± 0.12	50.4 ± 16.3	0.31 ± 0.33	0.20
Caudal abdomen	48.0 ± 17.4	0.16 ± 0.11	48.9 ± 17.0	0.24 ± 0.05	0.11

Observer 2					
	With dynamometer		Measuring tape alone		
Location	Girth (cm)	Precision of measurements (cm)	Girth (cm)	Precision of measurements (cm)	Difference in girth
	Mean ± SD	Mean SEM ± SD	Mean ± SD	Mean SEM ± SD	*p*-value
Middle neck	35.3 ± 10.3	0.29 ± 0.16	36.3 ± 10.9	0.41 ± 0.30	0.38
Cranial chest	60.8 ± 17.4	0.27 ± 0.22	60.8 ± 17.1	0.19 ± 0.12	0.69
Widest chest	58.8 ± 16.5	0.41 ± 0.21	59.7 ± 16.0	0.45 ± 0.33	0.11
Cranial abdomen	49.5 ± 16.6	0.34 ± 0.18	50.7 ± 16.3	0.39 ± 0.27	0.04
Caudal abdomen	49.1 ± 17.3	0.28 ± 0.18	48.7 ± 16.3	0.38 ± 0.23	0.31

SD, standard deviation; SEM, Standard error of mean. The table shows data from the subgroup of eight dogs. Wilcoxon signed rank test was used for comparisons between measurements performed with a measuring tape equipped with a dynamometer and a measuring tape alone, per observer, where the means of the triplicates were used in the analyses. A *p*- value of < 0.05 was considered significant.

#### Correlation between girth measurements performed with and without a dynamometer attached to the measuring tape

**Figure 2:**
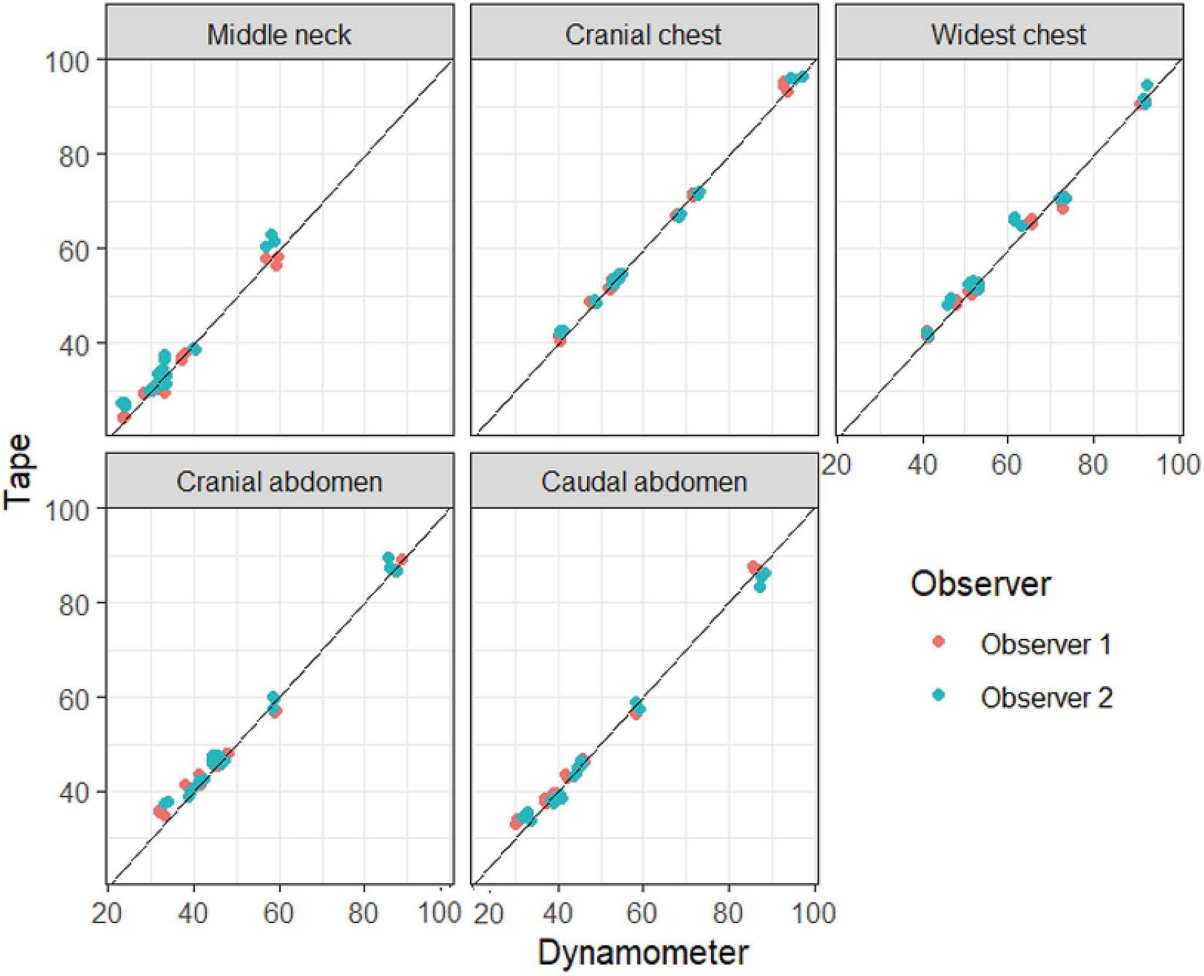
Visualization of the agreement between measurements performed with and without a dynamometer attached to the measuring tape. Girth measurements over the neck, chest, and abdomen were performed with a dynamometer attached to a measuring tape (*x*-axis, “dynamometer”) and with a tape measure alone (y-axis, “tape”). All triplicates (in centimeter) for the subgroup of dogs (*n* = 8) are shown in the plots, and the measurements of the two observers are color-coded. The lines represent a perfect agreement between the two methods (a slope of 1).

Pearson’s correlations (r) were calculated for girth measurements performed with a measuring tape equipped with a dynamometer compared to a measuring tape alone, per observer, with all anatomical locations pooled. The correlations between the two methods were high: 0.998 for observer 1 and 0.996 for observer 2 (*p* < 0.0001 for both). Figure 2 illustrates the almost perfect agreement between the two different methods, with the individual triplicates of each observer plotted per anatomical location.

## Discussion

In this study, dogs were evaluated using girth measurements made with a measuring tape, both with and without an attached dynamometer. This study aimed to assess intra- and inter-observer reliability of neck, chest, and abdominal girth measurements, as this method, if reliable, could be a valuable addition to clinical BCS assessment in dogs. Girth measurements performed with a dynamometer demonstrated high intra- and inter-observer reliability at all evaluated anatomical locations. The highest intra- and inter-observer reliability was observed for the cranial chest and caudal abdomen, while the lowest inter-observer reliability was observed for the middle neck. The anatomical location with the highest precision of measurements was the cranial chest, while the location with the lowest precision was the widest chest. Correlations between the girth measurements performed with and without a dynamometer attached to the measuring tape were high, but higher girth values with lower precision were recorded using only a measuring tape.

### Intra- and inter-reliability in the form of intraclass correlation coefficients

The ICC values for all anatomical locations, both within and between observers, were high (Intra-observer ≥0.967 and Inter-observer ≥0.985), according to previously defined ranges (3). To our knowledge, reliability studies of neck, chest, and abdominal girth measurements in dogs, for comparison of the results, are lacking. However, the reliability studies of waist circumference measurements in humans, similar to our findings, showed high intra- and inter-observer reliability (≥0.950) (34, 38). Compared to previous reliability studies of limb girth measurements in dogs (3, 4, 35, 39, 40), the ICC values of this study showed slightly to markedly higher reliability, which could be due to higher performance of the observers, a better measurement device, or that the measurement sites were easier to evaluate in this study.

#### Performance of the observers

The findings of this study indicated that the observers were consistent in their measurements and displayed strong agreement with one another. Even so, some differences existed between the two observers despite their uniform background experience and despite the standardization of measurements performed before the start of the study. Observer 2 recorded significantly higher girth values at two locations than observer 1. In addition, observer 2 exhibited numerically lower precision of measurements compared to observer 1, but the differences were not significant. Intra-observer reliability was high for observer 1 across all anatomical locations, while observer 2 showed slightly lower, but still high, intra-observer reliability. It has been demonstrated in a previous reliability study of limb girth measurements (3) that observers can perform measurements with varying levels of precision, even when their background experience is quite uniform. As could be expected, more experienced observers can show higher intra-observer reliability (4). At evaluation of abdominal obesity in humans using measuring tape in overweight and obese patients, measurements made by general practitioners were particularly inaccurate when compared to experts (36). During the measurement period of the 16 dogs in this study, an increase in the precision of measurements was indicated in both observers, shown by a numerically decreasing SEM. It is, therefore, likely that the observers, by increasing their experience during the current 4-month study period, improved their measurement and/or palpation techniques. The subgroup of dogs, which was measured at the end of the data collection period (Table 4), also showed a numerically lower SEM than the whole cohort of dogs (Table 2). In a study on the reliability of lower extremity circumference measurements in humans using measuring tape alone, all six observers showed significantly higher circumference values at their second measurement compared to their first (2). However, one study showed no improvement in reliability at the second evaluation, when four observers evaluated the circumference measurements of brachium, crus, and thigh in 20 dogs twice during the same day (3). The results indicate that the experience of the observers must be gained over time, and ideally, the inclusion of dogs in the subgroup should have been randomized from the start of the study.

#### Impact of measurement device

Besides the individual performance of the observer, differences in reliability between studies could depend on the type of measurement device used (39). Devices were comparable between this study and previous canine studies of trunk measurements, i.e., measuring tape equipped with a dynamometer (41, 42) or measuring tape alone (26, 27, 32). A spring dynamometer is attached to improve consistency in tape tension, thereby minimize measurement variation resulting from differences in applied forces and differences in soft tissue compression (35). However, a canine study that evaluated four types of measuring tapes, all equipped with different kinds of dynamometers, showed that two of the devices collected values that were significantly lower compared to the other two devices (39), despite the fact that all used dynamometers attached to the measuring tapes. Consistency in tape tension has been identified as a challenging task in repeated evaluations, even when using a dynamometer (2). In this study, few significant differences were shown between girth measurements performed with and without a dynamometer, indicating somehow equal performance of the observers, even at measurements with a measuring tape alone. However, only eight dogs were included in the subgroup, and those results should be interpreted with caution.

#### Conformation of measurement sites

Besides the application of an exact tape tension, the shape and size of the measurement site (34, 35) and variations in the placement of the measuring tape can also add to the variation (35). The limbs of dogs, in comparison to the chest and abdomen, have shapes that are more conical, and the measuring tape could therefore be more difficult to keep in the exact position around the limbs without slipping. This theory is strengthened by studies showing that the canine thigh, which is a highly conical measurement site, appears more difficult to measure precisely (3, 40) than the thoracic limb (4). In this study, the highest intra- and inter-observer reliability and precision were observed for the anatomical locations of the cranial chest and caudal abdomen, sites without conical shape and thus low risk of slipping of the measuring tape. Another highly relevant factor is that both locations have distinct, palpable, and adherent anatomical structures (the axilla and the cranial part of the pelvic bone) to relate to, enabling the exact same site to be repeatedly evaluated by both observers. Locations lacking palpable and adherent structures, such as the middle neck and the widest chest, showed the lowest inter-observer reliability and precision, probably due to increased variation in the placement of the measuring tape. In addition, the canine neck is conical in its conformation. If the location of the neck should be used for evaluation in a clinical canine patient, we recommend deciding on a certain proportion of the distance (2, 4), i.e., from the most caudal crest on the skull to the cranioproximal part of the scapula. Preferably, the distance should be marked with adhesive marking before girth measurements in triplicates to ensure the exact same measurement position. To determine the location of the widest part of the chest, viewed from above, a certain rib may be counted during the initial evaluation and used as a reference in the follow-up evaluations since the position of the widest chest area can vary in dogs that increase or decrease body condition (28).

The proportional size of the measurement error, i.e., the size of the recorded measurement error (SEM) in relation to the measured girth, is dependent not only on the size of SEM but also on the girth value of the site. The fact that the girth values of the neck, chest, and abdomen are all greater than the girth of the limbs of dogs could be one part of the explanation of the high ICC values of this study. However, the mean size of SEM in comparison to the mean girth value constituted a low proportional size for all anatomical locations evaluated (≤ 1%), which is in line with another study of girth measurements of the trunk in dogs (37). In humans, the proportional size of the measurement error of the waist circumference was higher in overweight and obese participants compared to regular weight participants (36, 38). Therefore, low precision might also be a potential risk when evaluating obese dogs with a prominent subcutaneous fat layer. Only one obese dog participated in this study. However, Witzel et al. (37) included only overweight to obese dogs in girth measurements of the chest and abdomen (37) and showed a proportional size of the measurement error just in line with this study, indicating that the method could also be used in obese dogs with reliable results.

#### Clinical applications

Studies on weight reduction in overweight dogs have shown that both chest and abdominal girth measurements decreased significantly after physical exercise, with or without caloric restriction (26, 27). Similarly, dogs recovering from decompressive surgery following thoracolumbar disk extrusion showed significant reductions in their abdominal girth measurements (41, 42). Chun et al. (32) evaluated the widest chest and caudal abdominal girths of Beagle dogs that gained weight (32). The chest girth differed by a mean of 7.8 cm, and the abdominal girth differed by a mean of 14.6 cm in underweight (BCS 3) compared to obese dogs (BCS 8). A study by Söder et al. (28) investigated 21 normal weight to slightly overweight dogs of small- to giant-sized breeds that exercised with their owners for 8 weeks (28). The dog cohort significantly decreased in mean BCS by 0.4 score (from 5.1 to 4.7). It significantly reduced the cranial chest girth (mean 2.5 cm) and the caudal abdominal girth (mean 1.4 cm) after the exercise program (28). It is thus evident that underweight compared to obese dogs of the same breed (32) may differ widely in their girth measurements. In contrast, statistically significant reductions of the chest and abdominal girths in merely slightly overweight dogs of different breeds may be as low as a few centimeters (28). A prioritized question would be to discuss the validity of a change in girth, i.e., enable distinction between valid girth changes and changes arising from measurement errors. To bring up such a discussion, calculations were carried out to explore the validity of a change in girth (Supplementary Table S1 in Supplementary File 2). The calculations were based on the results from the former study by Söder et al. (28)evaluating chest and abdominal girth measurements before and after an exercise program in dogs (28), combined with the obtained measurement errors (SEM) generated in this study, by inspiration from Wang et al. (38). According to the calculations carried out, the anatomical locations of the cranial chest and caudal abdomen, in the study by Söder et al. (28) showed changes in girth measurements that were of magnitude 2–5 times greater than the measurement errors of the repeated evaluation (Supplementary Table S1 in Supplementary File 2), supporting the validity of the changes.

The next question for discussion would be to reflect on whether a change that is statistically significant and considered valid (i.e., a difference greater than two times the mean of SEM) automatically impacts animal health, i.e., clinically relevant. In humans, the definition of what constitutes a clinically relevant change of the abdominal circumference has been proposed to be unclear (43). Differences exceeding 3.0 cm in waist circumference and 2.0 cm in hip circumference measured by the same observer have been proposed as thresholds for people, calculated from the measurement errors and the statistical significance of the method (38). However, compared to humans, dogs come in many different sizes and breeds, establishing thresholds of chest and abdominal girth measurements in dogs related to clinical relevance might be difficult. Nevertheless, in the context of overweight dogs that need to lose weight, a reduction in bodyweight of approximately 4–5% (30), equivalent to an average decrease of 0.5 BCS score (10), has been associated with significantly increased activity and quality of life (30). Such a reduction of overweight could suggest a clinically relevant change in dogs, which could be put about the change in girth. The dog cohort in the study by Söder et al. (28) showed a decrease of mean 0.4 BCS score (28), but were only normal weight to slightly overweight at baseline, making the clinical relevance of the BCS reduction not wholly comparable to cohorts of overweight to obese dogs making a similar reduction (30). To take this discussion further, girth measurements of the chest and abdomen could be evaluated in future studies of overweight dogs prescribed weight loss by caloric restriction and/or physical exercise to confirm whether detectable changes in girth measurements of the chest and abdomen could be considered valid with the proposed measurement technique of this study. Moreover, future studies should investigate to what extent the changes in girth associated with changes in BCS and the health and welfare of the animal, to explore the clinical relevance of chest and abdominal girth changes in dogs.

## Study limitations and future perspectives

The limitations of this study are several. No measuring of distances and/or markings of anatomical locations were used, which impedes the guarantee that the same location was repeatedly evaluated. Therefore, the variation recorded in this study could depend on differences in the placement of the measuring tape and not alone on differences in tape tension. In contrast, the proposed measurement technique in this study is quick and easy and should be achievable even under stressful clinical conditions. The dog population was relatively small, especially the subgroup of dogs. Ideally, all dogs should have been evaluated both with and without the dynamometer attached to the measuring tape. The dog population mimicked a clinical situation and was heterogeneous with variation in dog size and coat length, but despite this heterogeneity, the measurements showed high reliability. However, a slight difference in the cranial chest or caudal abdominal girths in a small dog might be associated with a more significant difference in BCS than in a large breed, which is a factor that needs to be evaluated in future studies about the validity and clinical relevance of the girth change. Additionally, only one underweight dog and one obese dog participated. Further reliability studies are recommended in overweight and obese dogs (BCS 7–9), preferably undergoing, e.g., weight reduction, as the measurement error might be higher in obese dogs as shown in people (36, 38). The recorded girth differences should be compared to BCS, in addition to total fat and lean mass percentages assessed by an advanced objective method (e.g., DEXA). The observers were both inexperienced evaluators, which precluded comparison with an experienced observer. Conversely, the results show that measurements with a measuring tape equipped with a dynamometer should be feasible for any veterinarian or veterinary nurse at the clinic after a short training period.

## Conclusion and clinical importance

Girth measurements proved reliable for all anatomical locations evaluated, but especially for the cranial chest and caudal abdomen that showed high precision within and between observers. A measuring tape equipped with a dynamometer is recommended, as measurements recorded with a measuring tape alone showed a tendency toward higher girth values with lower precision. Further reliability studies are recommended in overweight and obese dogs to confirm that the high reliability of the method also applies to this patient group. Future research should evaluate neck, chest, and abdominal girth measurements in overweight canine patients and investigate the usefulness of the method as a valuable addition to clinical BCS assessment following changes in body composition.

## Data Availability

The original contributions presented in the study are included in the article/Supplementary material, further inquiries can be directed to the corresponding author.
