# Supplementary material for: Intra- and inter-observer reliability of girth measurements of the neck, chest, and abdomen in dogs
Source: Front Vet Sci. 2025 Mar 13;12:1546951. doi: 10.3389/fvets.2025.1546951 (PMC11949135; doi:10.3389/fvets.2025.1546951)
Supplement: Supplementary file 2 [file Supplementary_file_2.docx]

**Supplementary file 2.** Calculations to explore the validity of a change in girth

The standard errors of mean (SEM), for observers 1 and 2 pooled, on the whole current dog cohort (n=16), were compared with the statistically significant changes in girth shown by Söder et al. (2024) for the cranial chest and the caudal abdomen (see calculations in Table S1). The evaluation of dogs in the study by Söder et al. (2024) was performed repeatedly, before and after an exercise programme. Therefore, the mean SEM values from the current study (for the locations of the cranial chest and the caudal abdomen) were multiplied with two and thereafter compared with the recorded mean difference in girth (Söder et al. 2024), for the respective location (Table S1). According to the calculations (Table S1), the magnitude of the differences were 2-5 times greater than the measurement error for a repeated evaluation, indicating the changes to be valid and not arising from measurement errors.

**Table. S1.** The calculations for exploring the validity of changes were based on results from a former exercise intervention study (Söder et al. 2024 ^†^), and on standard errors of mean (SEM) from the current study.

|  | **Söder et al. 2024** | **Observers current study** | | **Validity of change** | | |
| --- | --- | --- | --- | --- | --- | --- |
| **Location** | **Difference in girth before and after exercise**  (*cm)* | **Precision of measurements derived from triplicate**  *(cm)* | **Measurement error for repeated evaluation**  *(cm)* | **Difference in girth versus measurement error**  *(cm)* | **Magnitude of difference in relation to measurement error**  *(times)* |  |
|  | *Mean Diff* | *Mean SEM* | *2*SEM* | *Diff vs 2*SEM* | *Diff****/****2*SEM* |  |
| Cranial chest | 2.5 | 0.23 | 0.46 | 2.5 > 0.46 | ~ 5 |  |
| Caudal abdomen | 1.4 | 0.34 | 0.68 | 1.4 > 0.68 | ~ 2 |  |

^†^ Söder, J., Roman, E., Berndtsson, J. *et al.* Effects of a physical exercise programme on bodyweight, body condition score and chest, abdominal and thigh circumferences in dogs. *BMC Vet Res* **20**, 299 (2024). https://doi.org/10.1186/s12917-024-04135-3

Diff: Difference, SEM: Standard error of mean. The SEM presented in this table is the calculated mean SEM from observers 1+2 pooled, of the current study.
